# A Comprehensive Approach to Disentangle the Effect of Cerebellar Damage on Physical Disability in Multiple Sclerosis

**DOI:** 10.3389/fneur.2020.00529

**Published:** 2020-06-30

**Authors:** Serena Ruggieri, Komal Bharti, Luca Prosperini, Costanza Giannì, Nikolaos Petsas, Silvia Tommasin, Laura De Giglio, Carlo Pozzilli, Patrizia Pantano

**Affiliations:** ^1^Department of Human Neurosciences, “Sapienza” Rome University, Rome, Italy; ^2^Department of Neurosciences, San Camillo Forlanini Hospital, Rome, Italy; ^3^Department of Radiology, IRCCS Neuromed, Pozzilli, Italy

**Keywords:** multiple sclerosis, magnetic resonance imaging, cerebellum, physical disability, atrophy, diffusion tensor indexes

## Abstract

Cerebellar damage occurs frequently in multiple sclerosis (MS) patients, with a wide exhibition of symptoms particularly as impairments of balance and gait. Recent studies implementing new postprocessing magnetic resonance imaging (MRI) techniques showed how cerebellar subregional atrophy provides an explanation of disability in MS. The aim of this work was to evaluate the relationship between quantitative measures of physical disability, cerebellar subregional atrophy, and cerebellar peduncle disruption. Forty-nine MS patients and 32 healthy subjects as controls (HS) underwent a 3-Tesla MRI including 3D T1-weighted and diffusion tensor imaging. Patients underwent static posturography to calculate the body's center of pressure (COP) displacement, Expanded Disability Status Scale (EDSS), and 25-ft walking test (25-FWT). Cerebellar lobular volumes were automatically calculated using the Spatially Unbiased Infratentorial Toolbox. Tract-based spatial statistics (TBSS) in FSL was used to process diffusion tensor imaging (DTI) Fit-generated fractional anisotropy (FA) maps to assess structural connectivity of cerebellar peduncles. Stepwise multivariate linear regression analyses were used to explore relationships between variables. Cerebellar volumes (anterior and posterior, as well as lobular volumes from I to X) were significantly lower in patients with MS than HS (*p* < 0.05). FA in all cerebellar peduncles was lower in MS patients than in HS (*p* < 0.05). EDSS and 25-FWT showed an association with atrophy of lobule VIIIb (β = −0.37, *p* < 0.01, and β = −0.45, *p* < 0.001, respectively) COP measures inversely correlated with volume of lobules I–IV (β = −0.37, *p* < 0.01, and β = −0.36, *p* < 0.01). Lower FA in the three cerebellar peduncles of MS patients positively correlated with cerebellar lobular volumes. Our findings show how sensorimotor cerebellum atrophy and disruption of both afferent and efferent cerebellar connections contribute to physical disability in MS patients.

## Introduction

Cerebellar involvement has been included as a feature of multiple sclerosis (MS) patients ever since the disease was first described (1). Classically, Charcot referred to symptoms due to cerebellar damage as belonging to the well-known triad of “tremor, nystagmus and scanning speech.” Over the last century, an extensive body of evidence has shown how cerebellar functions can be impaired at various levels, from pure motor symptoms to cognitive-affective disturbances (2). Among physical symptoms, balance impairment is frequently reported in patients with MS and is described as one of the most disabling symptoms of this disease because it reduces independence and leads to falls and injuries (3).

Correlations between cerebellar pathology and commonly used measures of disability such as the Expanded Disability Status Scale (EDSS) have proved to be weak (4) or even absent (5, 6). This can derive from several aspects: the results of cerebellar structural magnetic resonance imaging (MRI) studies designed to assess disability outcome measures related to cerebellar alterations might vary considerably owing to the use of different MRI postprocessing techniques (7); cerebellar volume loss in MS patients may not be easily detectable using global segmentation or voxel-based morphometry techniques due to its morphological structural (2); measures of disability chosen might be too generic; indeed, when authors have explored associations between cerebellar volume and clinical outcomes by testing more specific skills, such as fine motor tasks (Nine Hole Peg test), ambulation [25-ft walking test (25-FWT)], or cognition (Paced Auditory Serial Addition Task), stronger results emerged (8, 9). Furthermore, a significant association between balance impairment and cerebellar functional and structural alterations has been reported (10, 11). The rate of balance impairment is reported to be linked to damage along specific white matter (WM) tracts as well as to cerebellar atrophy (10).

The latest innovative automatic cerebellar segmentation technique, which allows lobe-wise or even lobule-wise estimation of volumes (12), has shown to be more sensitive to cerebellar changes than conventional whole-brain voxel-based morphometry methods (13). Thus, the clinical impairment in MS, including various features of motor and cognitive disability, can be ascribed to cerebellar subregional atrophy being linked to alterations of the anterior and posterior cerebellar regions across different MS phenotypes (13, 14).

This aforementioned technique combined with more specific clinical measures of cerebellar dysfunction might improve our understanding of the clinical effects of cerebellar damage in MS. To our knowledge, the relationship between a quantitative measure of balance deficit and cerebellar lobular atrophy measured by using a segmentation technique based on a high-resolution atlas template has not previously been investigated. Additionally, the assessment of distinct feature of structural alteration involved in motor deficits can be exploited to plan research aimed to detect the individual patient's potential to restore central nervous system (CNS) functions in response to rehabilitation program.

Thus, the first aim of our study is to evaluate the specific contribution of cerebellar damage, in terms of lobular atrophy, to clinical impairment, including postural deficit, in patients with MS. Furthermore, besides assessing the grade of lobular cerebellar neurodegeneration, we aim to study the presence of tract-specific patterns of damage propagation to or from the cerebellum through its anatomical connections, to assess whether structural disconnections between the cerebellum and other parts of the CNS are related to cerebellar lobular atrophy.

## Materials and Methods

### Subjects and Clinical Assessment

This work was performed on a cohort of MS patients that has been described previously (15). Forty-nine patients with a diagnosis of MS according to the revised McDonald criteria (16) and 32 healthy controls (HS) were selected for the study. We reviewed all included patients' clinical history and confirmed the MS diagnosis according to more recent criteria (17).

To be included in the study, patients needed to fulfill the following inclusion criteria: age between 18 and 55 (inclusive) years; ability to walk without support/aid; and an EDSS score of between 0 and 5.5 (inclusive). Exclusion criteria were severe blurred vision, concomitant otological disease, significant cognitive impairment, relapse occurring over the previous 6 months, history of seizures, and contraindications to MRI. All the assessments (clinical evaluation, static posturography assessment, and MRI scan) were obtained within 1 week.

The study was conducted after institutional ethics committee approval and in accordance with the Declaration of Helsinki. Written informed consent was obtained from each subject before the start of the study procedures.

Demographic data were collected for each subject upon study entry. The body mass index (BMI) in the MS cohort was calculated as weight/height^2^ (kg/m^2^). MS patients underwent a detailed neurologic examination including the EDSS score and the 25-FWT (test covers 7.5 m; measured in seconds; longer performance time indicates greater disability) (18).

The assessment of static standing balance was performed according to standardized procedures, as published elsewhere (10, 15). Briefly, subjects were asked to stand barefoot on the ground, in an upright static condition, double-leg stance (standardized) and with arms resting at their sides on a using a force platform. Conditions of the room were standardized, and one experienced operator did all the evaluations. Displacements of the body's center of pressure (COP path) were recorded for 30 s in both the eyes open and eyes closed conditions (C-EO and C-EC, respectively) and evaluated at study entry (15).

### Imaging Protocol

Both patients and HS underwent an MRI scanning with 3-Tesla scanner (Magnetic Verio; Siemens, Erlangen, Germany) using a standardized protocol and a 12-channel head coil designed for parallel imaging (GRAPPA, generalized autocalibrating partially parallel acquisition). The following sequences were selected: a) dual turbo spin-echo [proton density (PD) and T2-weighted] [repetition time (TR) = 5,310 ms, echo time (TE) = 10/103 ms, echo train length = 28, matrix = 384 × 90, field of view (FOV) = 220 mm, iPAT reduction factor = 3], with 40 axial slices with 4-mm thickness and a 0-mm interslice gap; b) high-resolution three-dimensional T1-weighted (3D-T1) (TR = 1,900 ms, TE = 2.93 ms, 176 sagittal sections, 1-mm slice thickness, without gap, flip angle = 9°, FOV = 260 mm, matrix = 256 × 256); c) diffusion tensor imaging (DTI) (single-shot echo-planar spin echo sequence with 30 directions, TR = 12,200 ms, TE = 94 ms, FOV = 192 mm, matrix = 96 × 96, b = 0 and 1,000 s/mm^2^, 72 axial 2-mm-thick slices, no gap). We also acquired T1-weighted spin-echo images in patients alone after the administration of a gadolinium-based contrast agent (TR = 550 ms, TE = 9.8 ms, matrix = 384 × 90, FOV = 220 mm, time 2 min 15 s, with 40 slices with 4-mm thickness and a 0-mm interslice gap) to exclude acute inflammatory lesions that could influence further analysis.

### MRI Analysis

#### Lesion Volume

Lesion volumes were obtained by using a semiautomated technique on the basis of local thresholding by the Jim software (Jim, version 6; Xinapse Systems); lesions were segmented on PD images, whereas T2-weighted images were used to increase the confidence level in lesion identification. Lesion volumes yielded the following data for every subject: a quantification of the lesion burden total lesion volume (LV) and a binary lesion mask needed for the volumetric analysis, which was co-registered onto the 3D-T1 sequences.

#### Cerebellar Volume Analysis

For MS patients, 3D-T1 MR images were subjected to lesion filling in FSL (19). With the use of FLIRT (https://fsl.fmrib.ox.ac.uk/fsl/fslwiki/FLIRT), the core 3D-T1 MR images were first registered with the T2 MR images and then co-registered back to the lesion mask.

Lesion-filled 3D-T1 MR images of the MS patients and 3D-T1 MR images of the HS were segmented into gray matter (GM), WM, and cerebrospinal fluid (CSF) using the segmentation tool implemented in the Statistical Parametric Mapping (SPM12) software (http://fil.ion.ucl.ac.uk/spm). For each subject, intracranial volume (ICV) was computed as the sum of GM volume (GMV), WM volume (WMV), and CSF.

**Figure 1:**
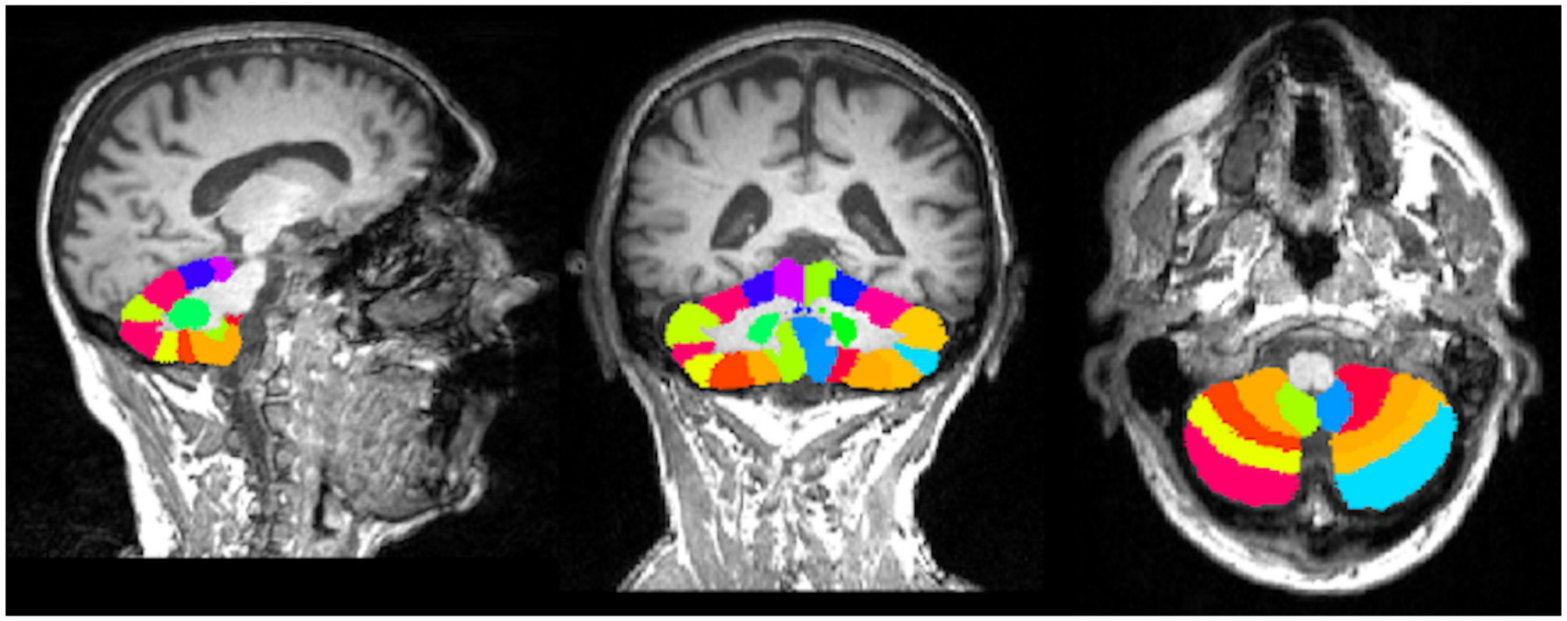
Spatially unbiased infratentorial toolbox (SUIT) cerebellar atlas aligned in the native subject space of individual multiple sclerosis (MS) patients.

To calculate the volumes of cerebellar lobules, we used the Spatially Unbiased Infratentorial Toolbox (SUIT) version 3.2 (http://www.diedrichsenlab.org/imaging/suit.htm) implemented in SPM12. The SUIT toolbox provides high-resolution atlas template for the cerebellum, which is unbiased and thus preserved the anatomical details of the cerebellar lobules (20). First, lesion-filled 3D-T1 images of the MS patients and core 3D-T1 images of HS were subjected to automatic segmentation and isolation to extract the cerebellum and its corresponding cerebellar mask. In the second step, we perform normalization to align the extracted cerebellum in the SUIT atlas template space, that is, the cerebellum template using the affine transformation matrix and the non-linear flow field. Every subject cerebellum was then resliced to modulate and preserve the cerebellar lobular volume in SUIT atlas template space (Figure 1). The lobular volume of the lesion-filled 3D-T1 images of the MS patients and 3D-T1 images of the HS were finally calculated, which were then compiled to obtain volume of anterior (I–V), posterior (VI–X), and whole cerebellum (hemispheres and vermis) (13).

#### Diffusion Tensor Imaging Analysis

DTI Fit, part of FMRIB's diffusion toolbox (http://fsl.fmrib.ox.ac.uk), was used to generate fractional anisotropy (FA) maps. The FA maps were subjected to the tract-based spatial statistics (TBSS) tool (https://fsl.fmrib.ox.ac.uk/fsl/fslwiki) for the voxel-wise statistical analysis and were then aligned into a common space using a non-linear image registration tool. The FMRIB58_FA_1 mm image was non-linearly registered with the other FA maps, which were then transformed into the Montreal Neurological Institute (MNI) standard space. The resultant of mean FA image was then thinned to create a mean FA skeleton, which was later thresholded at statistical power of 0.2. The output may act as a representative of the common center for all the WM tracts on which every subject-aligned FA maps were finally projected.

The processed DTI data using TBSS were taken at the next level of statistical analysis. Unpaired two-sample *t*-test was done for the voxel-wise general modeling and cross-subject statistics using the three cerebellar peduncles, that is, superior, middle, and inferior cerebellar peduncles (SCP, MCP, and ICP, respectively). The SCP, MCP, and ICP were selected as region of interest (ROI) using the JHU ICBMDTI-81 White-Matter Atlas inbuilt in FSL. The binarized masks of these three ROIs were transformed from the standard space to the native space using the combined linear and non-linear deformation matrices in FSL. For every individual, the average FA values of whole skeleton were strictly calculated within the binarized mask of SCP, MCP, and ICP. Later, the time series of the mean FA values for the whole skeleton was extracted within the binarized mask of SCP, MCP, and ICP using FSL meant command in FSL.

### Statistical Analysis

Analyses were carried out using a PC version of the Statistical Package for Social Sciences 22.0 (SPSS, Chicago, IL, USA). All values are presented as mean (standard deviation) or median [range], as appropriate.

The Shapiro–Wilk normality test was performed to check the normal distribution of data; variables showing a significantly asymmetric distribution (C-EO, C-EC, 25-FWT, and ICV) were normalized by log-transformation for the subsequent analyses. Differences between groups were calculated by using the unpaired two-sample *t*-test. Relationships between clinical variables were performed using Pearson's correlation coefficient (*r*) for continuous variables.

Partial correlations, corrected for age, gender, BMI, and ICV, were computed between each clinical measure of physical deficit and each MRI feature (supratentorial measures and lobular volumes). The false discovery rate (FDR) method was used to correct for multiple testing at the *p* < 0.05 level. Only variables with significant correlations were included in the following analysis. We further studied the relationships between clinical measures (EDSS, 25-FWT, C-EO, and C-EC) as dependent variables and GMV, WMV, LV, and each lobular volume from I–IV to X as independent variables, by stepwise multivariate linear regression analyses (for inclusion: *F* ≥ 1 and *p* ≤ 0.05; for exclusion: *F* < 1 and *p* > 0.05). Each model was adjusted for age, gender, BMI, and ICV. The latest was used as a covariate of no interest in the statistical analysis as a head-size normalization approach for both global and cerebellar measures.

Statistical analysis for the DTI parameter, that is, FA at the SCP, MCP, and ICP between MS patients and HS, was performed in FSL using a non-parametric test of 5,000 random permutations in a general linear model (GLM), and a threshold-free cluster enhancement technique (21) with covariates of no interest, that is, age and gender. The significant differences in the FA at the SCP, MCP, and ICP were corrected for multiple comparisons at the family-wise error (FWE) approach of *p* < 0.05. Lastly, the few-corrected FA differences at the SCP, MCP, and ICP were further correlated with the volumes of the cerebellar lobules in FSL. For this purpose, we again performed a non-parametric test of 5,000 random permutations in the GLM, with a threshold-free cluster enhancement technique (21) and covariates of no interest, that is, age and gender. The significant differences in the correlations findings were corrected for multiple comparisons at FDR of *p* < 0.05.

## Results

MS patients and HS did not differ in age and gender (*p* = 0.3 and *p* = 0.6, respectively). Demographic, main clinical variables, and MRI characteristics are summarized in Table 1.

**Table 1:** Clinical and MRI features for all subjects included in the analysis.

	**HS (*n* = 32)**	**MS (*n* = 49)**	*p*-value^*^
**Clinical variables**			
Mean age (SD), years	33.28 (7.6)	35.02 (8.1)	n.s.
Females/males	22/10	36/13	n.s.
Disease course, RR/SP	-	34/15	-
Mean disease duration, years (range)	-	7.87 (6.4)	
Median EDSS (range)	-	2.5 (1.0–5.5)	-
Mean 25-FWT, s (SD)		7.76 (3.1)	-
Mean C-EO, mm (SD)	-	441.76 (443.2)	-
Mean C-EC, mm (SD)	-	838.98 (845.3)	-
**MRI variables**			
WMV	417.95 ± 48.2	388.81 ± 51.1	0.044
GMV	704.95 ± 60.1	624.51± 63.8	0.011
LV	-	8.18 ± 8.1	-
Global cerebellar volume	119.59 ± 18.5	103.30 ± 13.1	0.039
Anterior cerebellar volume	14.98 ± 3.5	12.76 ± 2.3	0.036
Posterior cerebellar volume	104.61 ± 15.2	90.53 ± 11.8	0.034
Lobules I–IV volume	6.65 ± 1.5	5.47 ± 1.1	0.031
Lobule V volume	8.32 ± 2.0	7.28 ± 1.2	0.042
Lobule VI volume	19.43 ± 4.5	17.44 ± 2.2	0.047
Crus I volume	25.74 ± 5.3	22.39 ± 3.3	0.028
Crus II volume	19.06 ± 2.9	16.32 ± 2.9	0.026
Lobule VIIb volume	10.35± 1.2	8.74 ± 1.6	0.023
Lobule VIIIa volume	11.38 ± 1.3	9.74 ± 1.3	0.021
Lobule VIIIb volume	9.14 ± 1.2	8.74 ± 0.9	0.018
Lobule IX volume	7.73 ± 1.2	6.54 ± 0.9	0.015
Lobule X volume	2.15 ± 0.3	1.60 ± 0.3	0.013

*Volumes (in milliliters) are expressed as mean ± SD deviation. C-EO, displacement of body center of pressure with eyes open; C-EC, displacement of body center of pressure with eyes closed; EDSS, Expanded Disability Status Scale; GMV, brain gray matter volume; HS, healthy controls; ICV, intracranial volume; LV, whole brain lesion volume; MS, multiple sclerosis; WMV, brain white matter volume; RR, relapsing remitting; SP, secondary progressive*.

* *p-values for HC vs. MS comparison, false discovery rate (FDR) corrected*.

As expected, patients with greater disability in term of EDSS showed greater postural sway measured with C-EO and C-EC (*r* = 0.611 and *r* = 0.622, both *p* < 0.001, respectively) and greater 25-FWT score (ρs = 0.775, *p* < 0.001).

Compared with HS, MS patients showed lower GMV (*p* < 0.05) and WMV (*p* = 0.01). Accordingly, volume loss was observed in all the cerebellar lobules of MS patients than HS (all *p* < 0.05). The differences in the findings are summarized in Table 1.

Table 2 shows all partial correlations corrected for age, gender, BMI, and ICV. Higher EDSS and 25-FWT scores were associated with global atrophy as well-reduction in specific cerebellar lobular volumes. Similarly, measures of balance impairment display a correlation with supratentorial and cerebellar lobular degeneration (Table 2).

**Table 2:** Partial correlations between MRI features and measures of physical deficit, corrected for age, gender, BMI, and ICV.

	**EDSS**	**25-FWT**	**C-EO**	**C-EC**
WMV	−0.54^**^	−0.54^**^	−0.52^**^	−0.35^*^
GMV	−0.47^*^	−0.48^*^	−0.53^**^	−0.36^**^
LV	0.50^*^	0.20	0.39^*^	0.33^*^
Lobules I–IV	−0.38^*^	−0.44^*^	−0.49^**^	−0.42^**^
Lobule V	−0.40^*^	−0.50^*^	−0.45^*^	−0.32^*^
Lobule VI	−0.36^*^	−0.50^**^	−0.41^*^	−0.26^*^
Crus I	−0.25	−0.35^*^	−0.28^*^	−0.20
Crus II	−0.31^*^	−0.41	−0.32^*^	−0.30^*^
Lobule VIIb	−0.41^*^	−0.50^*^	−0.34^*^	−0.30^*^
Lobule VIIIa	−0.37^*^	−0.49^*^	−0.38^*^	−0.32^*^
Lobule VIIIb	−0.50^**^	−0.58^**^	−0.53^**^	−0.42^**^
Lobule IX	−0.34^*^	−0.41^*^	−0.35^*^	−0.28^*^
Lobule X	−0.27	−0.31	−0.28	−0.16

*25-FWT, 25-ft walking test; C-EO, displacement of body center of pressure with eyes open; C-EC, displacement of body center of pressure with eyes closed; EDSS, Expanded Disability Status Scale; GMV, brain gray matter volume; LV, whole brain lesion volume; WMV, brain white matter volume*.

* *p < 0.05*,

** *p < 0.01*.

The results of the stepwise multivariate linear regression analysis between clinical features and MRI parameters in the group of MS patients are summarized in Table 3 according to each model. The best model of each variable was characterized by the presence of lobule VIIIb for both EDSS and 25-FWT (β = −0.37, *p* < 0.01, β = −0.45 *p* < 0.001 respectively); while focusing on measures of balance, our data showed the predictive value of volume of lobules I–IV to explain both C-EO (β = −0.37, *p* ≤ 0.01) and C-EC (β = −0.36, *p* < 0.01) (Table 3). Lobule I–IV volume was the only independent predictor (*p* < 0.001) for both balance measures explaining 22 and 16% of the variance, respectively.

**Table 3:** Stepwise multivariate linear regression between clinical variables (EDSS, 25-FWT, C-EO, and C-EC) and MRI features in MS patients.

	**Model**				**Predictive variables**		
**Dependent variable**	***N***	**Adj *R*^**2**^**	***F***	***p***	**Variable**	**β**	***p***
EDSS	1	0.229	14.99	<0.001	LV	0.496	<0.001
	2	0.350		<0.001	LV	0.435	<0.001
					Lobule VIIIb	−0.368	<0.01
25-FWT	1	0.325	23.62	<0.001	Lobule VIIIb	−0.583	<0.001
	2	0.392	16.16	<0.001	Lobule VIIIb	−0.452	<0.001
					WMV	−0.310	<0.05
C-EO	1	0.217	14.01	<0.001	Lobules I–IV	−0.483	<0.001
	2	0.298	10.98	<0.001	Lobules I–IV	−0.424	<0.01
					WMV	−0.313	<0.05
	3	0.354	9.57	<0.001	Lobules I–IV	−0.375	<0.01
					WMV	−0.278	<0.05
					LV	0.267	<0.05
C-EC	1	0.158	9.84	<0.01	Lobules I–IV	−0.420	<0.01
	2	0.223	7.74	<0.01	Lobules I–IV	−0.360	<0.01
					LV	−0.289	<0.05

*Each model is adjusted for age, gender, body mass index and intracranial volume. EDSS, Expanded Disability Status Scale; 25-FWT, 25-ft walking test; C-EO, displacement of body center of pressure with eyes open; C-EC, displacement of body center of pressure with eyes closed*.

**Figure 2:**
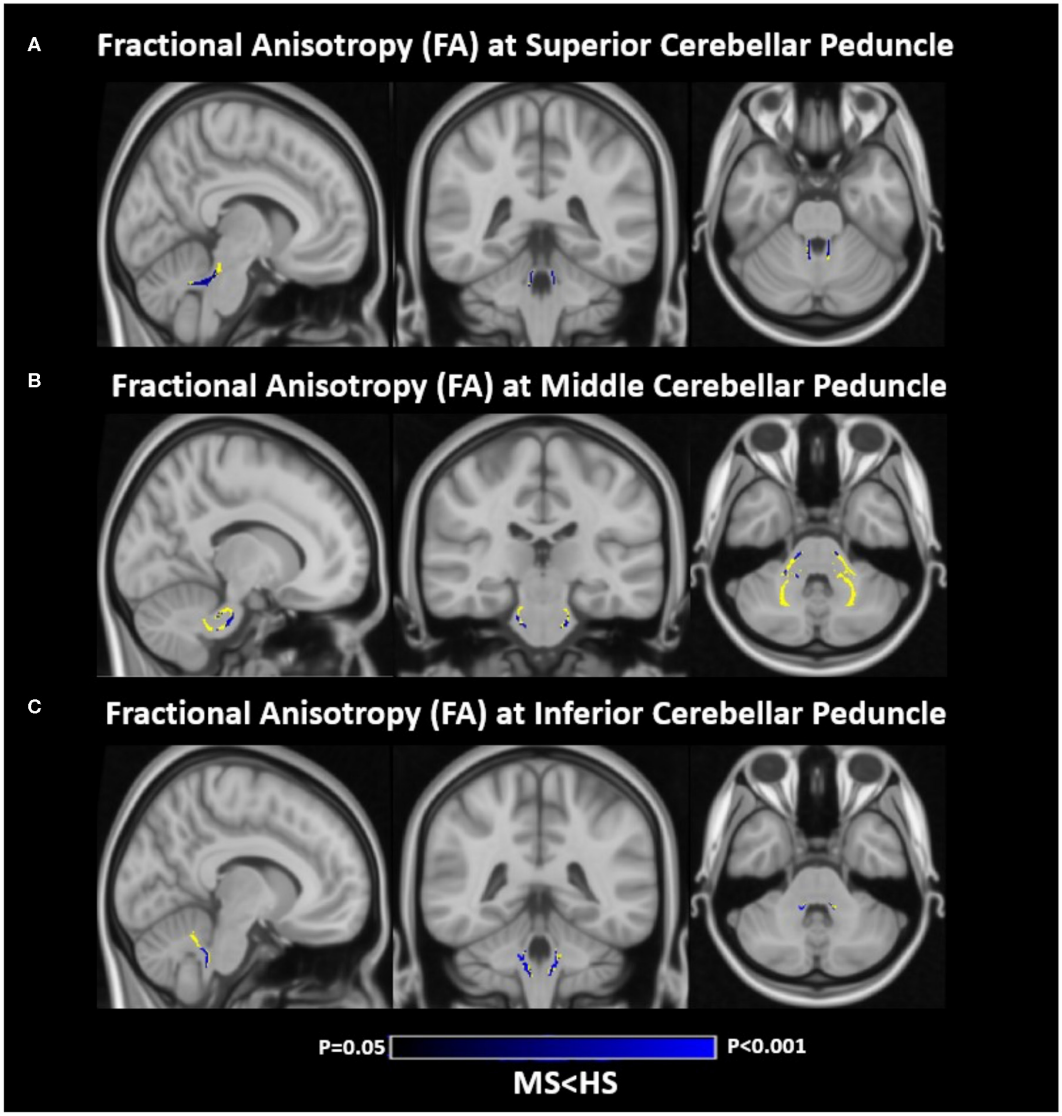
Differences of fractional anisotropy (FA) between the multiple sclerosis (MS) patients and healthy subjects (HS). **(A)** FA differences at the superior cerebellar peduncle (SCP). **(B)** FA differences at the middle cerebellar peduncle (MCP). **(C)** FA differences at the inferior cerebellar peduncle (ICP). Blue, decreased FA; yellow, binarized mask of SCP, MCP, and ICP. MS, multiple sclerosis patients; HS, healthy subjects. Results are corrected for multiple comparisons at family-wise error [(FWE) (*p* < 0.05)]. Images are shown according to radiologic orientation.

Lower FA (blue) was observed in MS patients than HS at all the three cerebellar peduncles, that is, SCP, MCP, and ICP (binarized mask; yellow) (Figure 2). Lower FA in all cerebellar peduncles of MS patients positively correlated with differences in volume of specific cerebellar lobules (SCP with lobules I–IV and VI; MCP with lobules I–IV, V, VI, VIIb, VIIIa, VIIIb, IX, crus I, and crus II; ICP with lobules VI and VIIIb; *p* < 0.05 for all correlations) as listed in Table 4.

**Table 4:** Correlations between fractional anisotropy (FA) indices at the three cerebellar peduncles and lobular cerebellar volume of MS patients.

**FA**	***R* value of cerebellar lobules**	***p*-value**	**1–*p* (FDR corrected)**	**Cluster size**	Peak *Z* stat^*^	**MNI coordinate**		
						***X***	***Y***	***Z***
SCP	I–IV	0.044	0.956	89	10	7.9	−54.1	−29.8
	VI	0.026	0.974	95	36	−2.6	−49.5	−23.1
MCP	I–IV	0.038	0.962	111	41	−14.5	−27.6	−35.9
	V	0.048	0.952	108	43	−14.9	−38.3	−35.0
	VI	0.028	0.972	113	32	−18.5	−35.0	−40.5
	VIIb	0.014	0.986	117	16	14.4	−32.4	−29.2
	VIIIa	0.039	0.961	110	10	13.3	−28.2	−33.4
	VIIIb	0.026	0.974	103	12	19.7	−54.8	−31.3
	IX	0.012	0.988	68	21	18.4	−47.4	−31.3
	Crus I	0.024	0.976	72	15	−27.5	−52.0	−37.4
	Crus II	0.048	0.922	118	14	−17.6	−54.8	−29.5
ICP	VI	0.018	0.982	108	27	9.15	−44.5	−34.0
	VIIIb	0.042	0.958	112	25	16.0	−29.9	−28.6

*Coordinates were extracted from Montreal Neurological Institute (MNI) 152 space. FA represents fractional anisotropy differences in multiple sclerosis patients at superior cerebellar peduncles (SCP), middle cerebellar peduncles (MCP), and inferior cerebellar peduncles (ICP). Direction of correlation represents positive correlation between lower FA differences at the three cerebellar peduncles and volumes of cerebellar lobules. Anatomical localizations of peak coordinates were established according to JHU ICBM-DTI-81 white-matter labels atlas and JHU white-matter tractography atlas. Results are corrected for multiple comparisons at a false discovery rate (FDR) of p < 0.05*.

* *Peak Z-stat denotes the maximum statistical value (Z-stat) for the peak activity*.

## Discussion

To the best of our knowledge, this is the first study that has explored the contribution of lobular cerebellar volume to physical disability in MS, including balance deficit. Our findings point toward the marked involvement of the sensorimotor cerebellum (anterior cerebellum and lobule VIIIb) in explaining physical deficits in terms of both gait and balance features.

In keeping with the findings of previous studies (6, 13, 14, 22), we observed severe damage of the cerebellum in MS, as shown by a significantly smaller cerebellar lobular and global volumes in MS patients than in HS.

Moreover, in line with previous works (15, 23), we have found a strong correlation between clinical measures of physical deficit and both supratentorial and cerebellar neurodegeneration. However, when we explored the contribution of MRI parameters among others, we found a significant association between volume of lobule VIIIb with EDSS and 25-FWT scores, whereas previous works have reported contrasting results, ranging from the absence of any significant association of cerebellar volume with EDSS (14) to a quite robust correlation (13). Conversely, walking speed as measured by 25-FWT correlated more consistently with cerebellar measures across literature (14, 24).

By using a relatively new automated technique, which allows the cerebellar individual lobules to be segmented and have been developed during the last years (12) and implemented in MS field recently, we have more specifically localized lobules I–IV as those implicated in balance alteration in MS. In a previous study of our group, voxel-based morphometry showed clusters of significant correlation between GM volume and balance performance, which were more widespread in cerebellar vermis (lobules IV, V, and VI), left and right cerebellar hemispheres (lobules IV, V, VI, and VIII), right crus I, and left and right lingual gyri (10).

Results from the present study highlight the functional dichotomy between the anterior lobe (lobules I–IV) and lobule VIIIb, which are known to constitute the sensorimotor topographical circuit of the cerebellum, whereas the posterior lobules contribute prevalently to cognitive processes (25).

We may further interpret our findings on mainly driven locomotion clinical measures (EDSS, 25-FWT) and balance measures (COP) as mirroring changes in the whole sensorimotor physiological functional organization of the cerebellum. Indeed, according to the most recent theory of cerebellar damage, every structure in the cerebellum is likely to play an important role in controlling and adjusting balance and locomotion, although each in a different way (26). Our results are in line with lesion-symptom mapping studies revealing that postural sway and altered balance control during gait were associated with lesions affecting the medial and intermediate zones of the cerebellum, especially in the anterior lobe (27).

With regard to cerebellar structural connectivity, here, we evidenced how microstructural alterations of cerebellar peduncles, frequently observed in patients with MS (24, 28), are strongly and specifically associated with cerebellar lobular atrophy. Indeed, our results revealed a strong association between cerebellar lobular atrophy and a reduction in FA in all three cerebellar peduncles. Interestingly, the strongest correlation between the diffusivity indexes and lobular volumes appears to follow the anatomical pathway of peduncle connections. Thus, disruption measured as FA of the SCP, which includes both afferent signals to the cerebellum (i.e., ventral spinocerebellar tract) and efferent axons from deep cerebellar nuclei to the brainstem and thalamus (29), which are involved in motor control, is strictly linked to atrophy of the anterior cerebellum. Conversely, abnormalities detected at MCP, which is the largest of the three cerebellar peduncles and includes afferent fibers that originate in the brainstem nuclei and contribute to the cortico-ponto-cerebellar pathway (29), were extensively associated across several cerebellar lobules. Lastly, the FA reduction at ICP, which includes the same proportion of afferent and efferent axons and connects the medulla to the cerebellum (29), is more closely correlated with inferior–posterior cerebellar atrophy. Although several factors very likely impact tissue loss in the cerebellum (2), secondary degenerative processes due to neural pathway disconnections caused by damage accumulation in distant sites might also play a prominent role.

These observations, combined with previous findings revealing (i) a significant correlation between DTI values in infratentorial WM tracts and measures of balance impairment (10) and (ii) SCP and MCP damage as contributing to ambulatory impairment in MS (28), lend further support to the hypothesis of impaired central integration of different inputs as a cause of physical disability in MS.

Advances in MRI techniques in recent decades allow researchers to define MS as a “disconnection syndrome” leading to cognitive deficit (30). This concept may be translated to physical disability. Indeed, the altered integration of neural pathways at different levels caused by the widespread, variable distribution and diverse nature (inflammatory and/or neurodegenerative) of CNS damage in patients with MS impairs ambulation, postural control, and the ability to maintain an adequate balance.

One limitation of our study is the relatively small number of patients included. The sample size, however, was large enough to support the concept that both cerebellar atrophy and disconnection are involved in sensorimotor deficits in MS. Another point to underline is that the involvement of brainstem and the spinal cord was only indirectly considered as fiber loss in the three cerebellar peduncles. The role of spinal cord in sensorimotor control is well-known, and atrophy of spinal cord has demonstrated to play a role in balance alterations in an extensive previous work (15). We also underlined that cerebellar lobular atrophy accounts only for a part of the variance of clinical measures explored, because certain damages in other structures of the CNS contribute to sensorimotor impairment as has been diffusively explored (15, 31, 32). We indeed aimed to explore the prevalent contribution of cerebellar degeneration to physical disability, trying to understand its interplay with the involvement of supratentorial GM and WM as well-lesion burden. However, we did not include the specific evaluation of deep GM volumes, even though it might be valuable given their fundamental role in conveying and routing sensory and motor signals among CNS and their established correlation with clinical disability (33).

Although our study has been assessed on MRI scan already acquired for a previous project, we used a 3D-T1 MRI sequence with 1-mm slice thickness, which is optimal to explore a small structure as cerebellum. Lesion volume has been assessed on 4-mm T2 axial images, which could be suboptimal compared with other sequences more sensitive for MS plaques (fluid-attenuated inversion recovery and double inversion recovery). However, given the impact of MS lesion volume evaluation on our imaging analysis, we considered a good standard of reference to use axial T2-weighted images.

Eventually, during the last years, the great interest regarding cerebellum involvement across different neurological diseases has been confirmed by the growing body of methodology to achieve a fine segmentation of this tiny structure (34). However, even though some newer cerebellar segmentation methods have shown higher accuracy (35) than SUIT, the latest has extensively proved to be valid in demonstrating differences between patients with MS and controls, as evidenced by a series of works published in the field (13, 14, 36), with the advantage of being more user friendly and less time-consuming (35). Despite these limitations, our findings indicate that regional atrophy in the cerebellum along with disruption of afferent and efferent pathways to this structure encompasses a pathological substrate of physical deficits seen in patients with MS and evaluated by measures of balance, EDSS, and 25-FWT. Cerebellar volume loss appears to be closely associated with microstructural alterations and to follow a tract-propagation pathway. These results shed light on our understanding of physical disability in MS and its neuropathological background and may help researchers to develop personalized rehabilitation program in MS patients, identifying exercise-induced changes in specific structures of cerebellum linked to disability or offering a substrate for future functional MRI studies to explore the plasticity of these recognized region of cerebellum (i.e., lobules I–IV and lobule VIIIb) in response to designed training.

## Data Availability Statement

The datasets generated for this study are available on request to the corresponding author.

## Ethics Statement

The studies involving human participants were reviewed and approved by Comitato Etico Policlinico Umberto I. The patients/participants provided their written informed consent to participate in this study.

## Author Contributions

SR and KB organized the dataset, were involved in the analysis and interpretation of data for the work, and wrote the first draft of the manuscript. LP and LG acquired the data. LP, CP, and PP contributed to the conception and design of the work. NP and CG acquired the data and contributed to the analysis of data. ST and LG contributed to the analysis of data and wrote a section of the manuscript. All authors contributed to manuscript revision, read, and approved the submitted version.

## Conflict of Interest

The authors declare that the research was conducted in the absence of any commercial or financial relationships that could be construed as a potential conflict of interest.
